# Combination Drug Use and Risk for Fetal Harm

**Published:** 2011

**Authors:** Wei-Jung A. Chen, Susan E. Maier

Alcohol and other drugs are frequently used in combination (Martin 2008; National Institute on Alcohol Abuse and Alcoholism 2008). Based on data from the National Epidemiologic Survey on Alcohol and Related Conditions, Falk and colleagues (2006, 2008) reported that 21.7 percent of the sampled population used both alcohol and tobacco and 5.6 percent used alcohol and another drug. Among women aged 18 to 24 the rates were 25.5 percent and 12.5 percent, respectively. Individually, alcohol, tobacco products, and a number of illicit drugs (such as cocaine or amphetamine) are known to be harmful to the developing fetus during pregnancy. Determining the additional harm resulting from polydrug use during pregnancy is an exceptionally challenging task. The unpredictable interactive (either additive or synergistic) effects of the drugs used simultaneously have far-reaching implications on child health and development given the pervasive use of multiple drugs in our society.

Changes in how drugs are absorbed, distributed, metabolized, and eliminated (i.e., pharmacokinetics) may help explain why polydrug use is dangerous to fetal development. Pharmacokinetic interaction is the process that occurs when two or more drugs are in the system at the same time. Although the pharmacokinetics of individual drugs may be well characterized, when the drugs are combined, one drug can seriously and unpredictably alter the concentration, bioavailability (the rate of a drug entering the bloodstream), and net effect of the other drug. Alternatively, the combination of drugs can alter the bioavailability of either or both drugs or form a metabolite more toxic than either of the parent compounds. For example, several well-known over-the-counter medications, such as aspirin, Tagamet^®^ (cimetidine), and Zantac^®^ (ranitidine), interact with alcohol metabolism leading to a higher level of blood alcohol concentration (BAC) (Baraona et al. 1994; Fraser 1998; Gentry et al. 1999). BAC levels for a given dose of alcohol are known and predictable; when another drug is added, alcohol metabolism is altered in an unpredictable manner. Given that BAC is a reliable predictor of the severity of alcohol-mediated brain injury in preclinical studies (Bonthius and West 1988, 1990), any drug that interferes with alcohol metabolism and results in an increase in BAC may be a potential cofactor in increasing alcohol-mediated damage.

In another example of the effects of combined drug use, Johnson and colleagues (1991) showed that cigarette smoking significantly reduced peak BAC in humans, and recent preclinical studies have reliably reproduced this finding (Gilbertson and Barron 2005; Parnell et al. 2006). Reduced BAC under the influence of nicotine presents a conundrum. Although decreasing BAC in the presence of nicotine may suggest a smaller injurious effect from alcohol, if someone desires to experience the “high” from alcohol or to drink to the point of inebriation, this decrease in BAC may promote additional alcohol use. This in turn may lead to an accumulation of acetaldehyde, an active and toxic metabolite of alcohol that exerts further damage to the physiological system, including the developing fetus.

As a final example, the interaction of alcohol and cocaine has been shown to be more harmful than the use of each drug individually because of the formation of the highly toxic metabolite cocaethylene (McCance et al. 1995; Pennings et al. 2002). Cocaethylene is an active metabolite of cocaine (Faroog et al. 2009; Patel et al. 2009), and it may account for the prolonged euphoria that occurs after concurrent use of alcohol and cocaine (McCance-Kate et al. 1998). Although clinical data regarding the negative effects of cocaethylene on fetal development are limited, the surviving offspring of mothers co-abusing alcohol and cocaine have shown neurobehavioral deficits (Singer et al. 2000). Moreover, preclinical studies using neonatal rats have shown toxic effects of the administration of cocaethylene on brain development (Chen and West 1997).

Potential harm to the developing fetus resulting from polydrug use during pregnancy is an important area of drug abuse research. Exploring the effects of each drug alone on the developing fetus does not capture the essence of the clinical condition. Studies on interactive effects of polydrug use fill a void in the scientific literature and highlight the importance of recognizing polydrug use during pregnancy as a significant maternal risk factor for fetal and child development and health. Research on how alcohol interacts with other drugs and how such interactions may adversely affect the developing brain will lead to a better categorization of the known detrimental effects from gestational polydrug use and a more focused understanding of the methods to avert or treat the outcomes.
